# Multilevel impacts of a pediatric early warning system in resource-limited pediatric oncology hospitals

**DOI:** 10.3389/fonc.2022.1018224

**Published:** 2022-10-12

**Authors:** Emily Mirochnick, Dylan E. Graetz, Gia Ferrara, Maria Puerto-Torres, Srinithya R. Gillipelli, Paul Elish, Hilmarie Muniz-Talavera, Alejandra Gonzalez-Ruiz, Miriam Armenta, Camila Barra, Rosdali Diaz-Coronado, Cinthia Hernandez, Susana Juarez, Jose de Jesus Loeza, Alejandra Mendez, Erika Montalvo, Eulalia Penafiel, Estuardo Pineda, Asya Agulnik

**Affiliations:** ^1^ The Chicago Medical School, Rosalind Franklin University of Medicine and Science, North Chicago, IL, United States; ^2^ Department of Global Pediatric Medicine, St. Jude Children’s Research Hospital, Memphis, TN, United States; ^3^ School of Medicine, Baylor College of Medicine, Houston, TX, United States; ^4^ Rollins School of Public Health, Emory University, Atlanta, GA, United States; ^5^ Pediatric Oncology, Hospital General de Tijuana, Tijuana, Mexico; ^6^ Pediatric Oncology, Hospital Dr. Luis Calvo Mackenna, Santiago, Chile; ^7^ Pediatric Oncology, Instituto Nacional de Enfermedades Neoplásicas, Lima, Peru; ^8^ Pediatric Oncology, Hospital Infantil Teletón de Oncología, Querétaro, Mexico; ^9^ Pediatrics, Hospital Central Dr. Ignacio Morones Prieto, San Luis Potosí, Mexico; ^10^ Pediatric Oncology, Centro Estatal de Cancerología, Xalapa, Mexico; ^11^ Pediatric Critical Care, Unidad Nacional de Oncología Pediátrica, Guatemala City, Guatemala; ^12^ Pediatric Critical Care, Hospital Oncológico Solca Núcleo de Quito, Quito, Ecuador; ^13^ Pediatric Oncology, Instituto del Cáncer Solca Cuenca, Cuenca, Ecuador; ^14^ Pediatric Oncology, Hospital Nacional de Niños Benjamín Bloom, San Salvador, El Salvador

**Keywords:** Pediatric Early Warning System (PEWS), pediatric oncology, global health, quality improvement, resource-limited, Latin America, pediatric critical care

## Abstract

**Background:**

Pediatric Early Warning Systems (PEWS) reduce clinical deterioration, improve interdisciplinary communication, and provide cost savings; however, little is known about how these impacts are achieved or related. This study evaluates the multi-level impacts of PEWS in resource-limited pediatric oncology centers.

**Methods:**

We conducted 71 semi-structured interviews including physicians (45%), nurses (45%), and administrators (10%) from 5 resource-limited pediatric oncology centers in 4 Latin American countries. Interviews were conducted in Spanish, transcribed, and translated into English. A code book was developed using *a priori* and inductively derived codes. Transcripts were independently coded by 2 coders, achieving a kappa of 0.8-0.9. Thematic content analysis explored perceived impacts of PEWS at the level of the *patient*, *clinician*, healthcare *team*, and *institution*.

**Results:**

PEWS improved the quality of attention for *patients*, reducing morbidity and mortality. *Clinicians* felt more knowledgeable, confident, and empowered providing patient care, resulting in greater job satisfaction. PEWS affected *team* dynamics by improving interdisciplinary (ward and intensive care unit) and interprofessional (physicians and nurses) relationships and communication. This ultimately led to *institutional* culture change with emphasis on patient safety, collaboration with other centers, and receipt of institutional awards. Together, these impacts led to hospital-wide support of ongoing PEWS use.

**Conclusions:**

In resource-limited hospitals, PEWS use results in multi-level positive impacts on *patients*, *clinicians*, *teams*, and *institutions*, creating a feedback loop that further supports ongoing PEWS use. These findings can guide advocacy for PEWS to various stakeholders, improve PEWS effectiveness, and inform assessment of other interventions to improve childhood cancer outcomes.

## Introduction

Hospitalized pediatric oncology patients are at high risk for clinical deterioration, particularly in resource-limited settings (1, 2). Pediatric Early Warning Systems (PEWS) are bedside assessment tools associated with an action algorithm used for early identification of patients at risk for deterioration (3), and have been validated to predict clinical deterioration in hospitals of all resource levels (4–7).

The impacts of PEWS have been demonstrated across multiple levels of hospital care. PEWS decrease clinical deterioration events and pediatric intensive care unit (PICU) utilization (8), and improve the perceived quality of care (9). PEWS have also been shown to foster nursing empowerment and increase confidence in recognizing and managing clinical deterioration (10), improve interdisciplinary communication and relationships (11, 12), and lead to cost savings (13).

While many positive impacts of PEWS have been identified in resource-limited settings, little is known about how these effects are achieved or interrelated. This study explores hospital staff perceptions of the multilevel impacts of PEWS, how they are achieved, and the process by which they facilitate and augment one another.

## Methods

This is a secondary analysis of a study designed to identify barriers and enablers to PEWS implementation, and study methods have been previously described in detail (14). The study was approved by the St. Jude institutional review board as a minimal risk and thereby exempt study. Additional approvals were obtained by participating facilities as required. Written participant consent was waived on account of the study’s exempt status; each participant provided verbal consent prior to the start of their interview. The Consolidated Criteria for Reporting of Qualitative Research (COREQ) guidelines were used to maintain rigor of qualitative reporting (15).

### Hospital and participant selection

Escala de Valoraciόn de Alerta Temprana (EVAT) is a Spanish-language PEWS validated in pediatric oncology patients (4). Proyecto EVAT is an international collaborative led by St. Jude Children’s Research Hospital (St. Jude) to support PEWS implementation in resource-limited hospitals providing pediatric oncology care in Latin America (16, 17).

Five Proyecto EVAT centers which completed PEWS implementation prior to March 2020 were selected to participate in the study with representation from Mexico, Central America, and South America. Center characteristics are described in **Supplemental Table 1**. Each center selected a study lead who identified 10-15 participants involved in PEWS implementation, including PEWS implementation leaders, hospital administrators, and staff indirectly involved in utilizing PEWS.

### Data collection

An interview guide (**Supplemental Figure 1**) was designed to identify barriers and enablers to PEWS implementation at participating centers (14). The guide was translated to Spanish, iteratively revised for relevance and clarity, pilot tested with three individuals from hospitals not participating in this study but demonstrative of the target population, and modified based on feedback. Interviews were conducted in participants’ native language (Spanish) by bilingual members of the research team (PE, SG) *via* a video conferencing platform (WebEx) from June to August of 2020. The interviewers were previously unknown to participants, not affiliated with their hospital, and not involved in PEWS implementation. Audio recordings of the interviews were professionally transcribed, translated to English, and de-identified (removing all names and other identifiers) prior to analysis.

### Analysis

A codebook was established using *a priori (* 18) along with inductively-derived codes defined by two authors (AA, GF) through iterative review of nine transcripts. Two authors (AA, GF) independently coded each transcript using MAXQDA software (VERBI GMBH, Berlin, Germany), achieving a kappa of 0.8-0.9. Incongruities in coding were resolved by a third author (DG) serving as an arbitrator.

Three “outcomes” codes were identified to describe perceived impacts of PEWS at the level of the *patient*, *individual*, and *institution* (**Supplemental Table 2**). *Individual* outcomes were subsequently split into impacts on the *clinician* and healthcare *team*. Thematic content analysis explored participant perceptions of these multilevel impacts of PEWS at their centers. Codes were examined independently and concurrently with constant comparative analysis of transcripts by site, participant role (e.g., clinician vs. non-clinician and nurse vs. physician), and center characteristics (e.g., presence or absence of a dedicated PICU).

## Results

**Figure 1:**
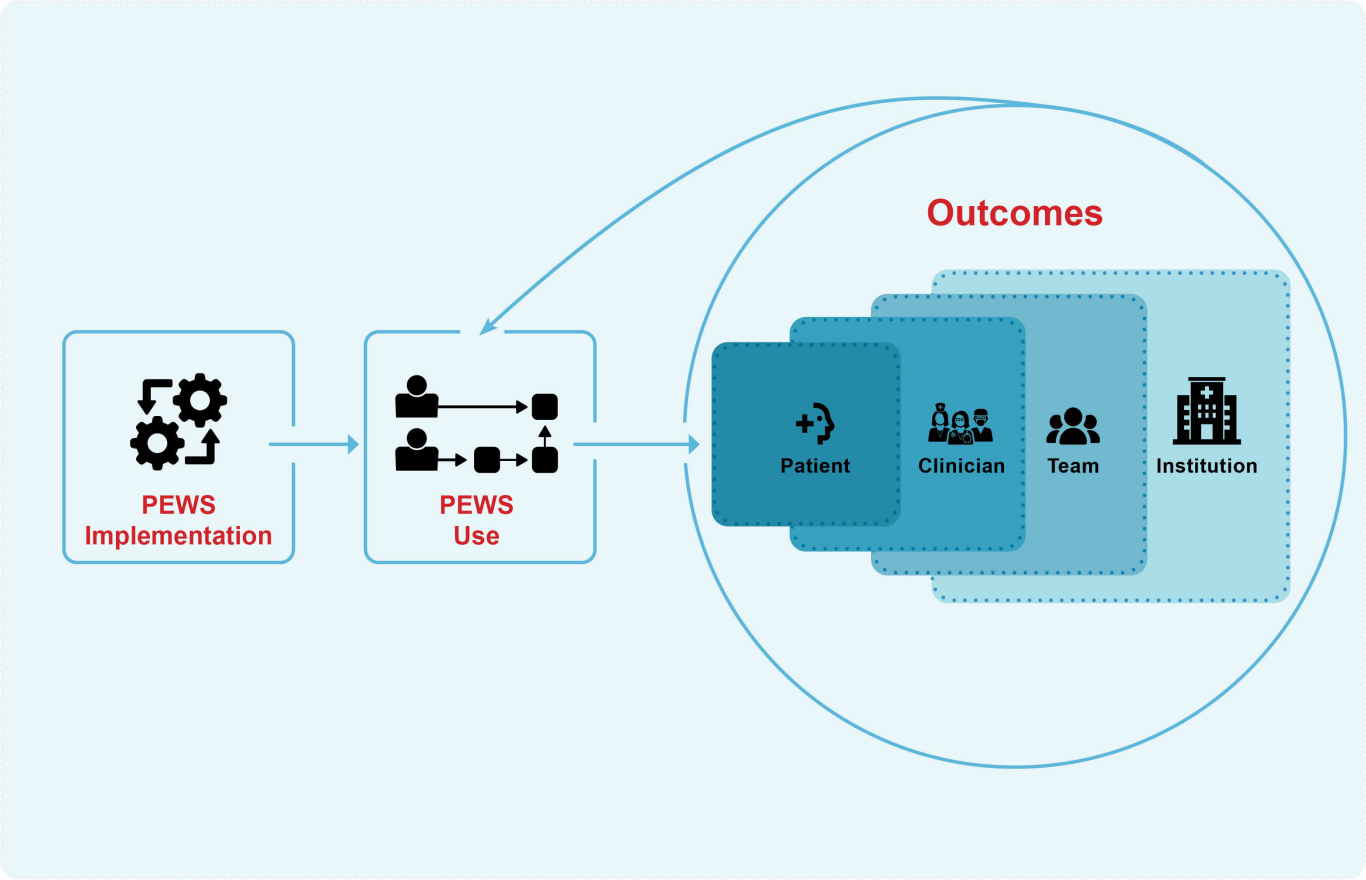
PEWS Cycle of Reinforcement. This figure describes staff perception of the impact of PEWS use on patients, clinicians, teams, and institutions. The benefits at each of these levels facilitate, augment, and reinforce the positive outcomes at the other levels and support ongoing PEWS use.

Seventy-one interviews were conducted at 5 pediatric oncology centers in Latin America (see **Table 1** for participant characteristics). Content analysis revealed perceived benefits of PEWS for *patients*, *clinicians*, *team* dynamics, and *institutions*. **Figure 1** summarizes these multilevel effects and their interplay, which is further described below.

**Table 1:** Characteristics of interview participants.

Characteristic		n	%
**Center**			
	Lima, Peru	18	25.4%
	San Luis Potosi, Mexico	11	15.5%
	San Salvador, El Salvador	15	21.1%
	Cuenca, Ecuador	15	21.1%
	Xalapa, Mexico	12	16.9%
**Profession**			
	Ward Physician	26	36.6%
	ICU Physician	6	8.5%
	Nurse	32	45.1%
	Other	7	9.9%
**Gender**			
	Male	21	29.6%
	Female	50	70.4%
**Years working in center**			
	0-10	27	38.0%
	11-20	25	35.2%
	21+	19	26.8%
**Role in hospital**			
	Administrator	8	11.3%
	Clinician	30	42.3%
	Clinician-Director	33	46.5%
**Role in PEWS Implementation**			
	Implementation Leader	39	54.9%
	Director	21	29.6%
	Other	11	15.5%
**Total**		**71**	**100.0%**

Adapted from Agulnik et al. (14).

### Patient

Participants at all centers described similar benefits of PEWS for patients including higher-quality patient attention, earlier detection of deterioration, and a reduction in morbidity and mortality (**Table 2**).

**Table 2:** Patient outcomes.

Higher Quality Patient Attention	Attention for the patient according to their disease, the kind of risks they have (Nurse, Xalapa)
	Improving the quality of attention for the hospitalized oncology patient has been the main thing (Ward Physician, San Salvador)
	The attention is faster and more precise (Nurse, Cuenca)
Early Detection & Prevention of Deterioration	[PEWS] allows us to control and monitor the patient before deterioration … before the vital functions are too late to act (Ward Physician, Lima)
	Children’s health conditions were deteriorating and we didn’t know until they were in critical condition, but with [PEWS] everything changed … we don’t wait until it’s too late (Nurse, Cuenca)
	We’re able to capture the probability for this patient to get critical in the next few hours and everything we must do to prevent this (Ward Physician, San Salvador)
Reduced Morbidity & Mortality	The most important thing was the reduction of the morbimortality of the patient, that has been very visible. (Ward Physician, Xalapa)
	Mortality has decreased by 2/3. (ICU Physician, San Salvador)
	We saw a decrease of adverse events and complications (Nurse, San Luis Potosi)

Using PEWS required frequent and focused patient assessments leading to increased situational staff awareness and more individualized attention: “*we’re not only applying a routine on that patient, there was more specific care depending on their current situation,”* (Nurse, Xalapa). Staff explained that, prior to PEWS, deteriorating patients would go undetected for hours as their bed was the last discussed on rounds or because vital signs were checked only once or twice a shift: *“before when a child got critical in the [ward] no one was aware of him”* (Ward Physician, San Luis Potosi). With the PEWS algorithm, patients were monitored at appropriate intervals based on their clinical status (**Supplemental Figure 2**). Thus, staff were better able to track the condition of each patient and focus their attention and resources where they were most needed: *“they enter the service and the first thing they do is check the sheet and see if someone has a red or yellow [PEWS] so they can start to work on that patient”* (Nurse, Lima); *“now, the detection of the child is done on time”* (Ward Physician, San Luis Potosi).

Greater situational awareness facilitated early detection of clinical deterioration and increased opportunities for prevention of critical illness: *“if you see a patient who doesn’t look that bad but he has a yellow [PEWS], it makes you act before; you prevent a bigger complication and it doesn’t depend on what you see but it’s something more objective”* (Ward Physician, Lima). As a result, deteriorating patients were identified and treated earlier, leading to fewer unplanned PICU transfers. Patients who did need PICU care were transferred earlier and required fewer interventions: *“The patient doesn’t need to go to intensive therapy to get better. In case the patient goes to intensive therapy … he won’t stay too long or need a tube,”* (Nurse, San Luis Potosi).

These improvements led to a perceived reduction in the morbidity and mortality of hospitalized patients: “*the mortality was highly reduced*,” (Ward Physician, San Luis Potosi). Additionally, with early detection, a patient transfer to the PICU was no longer synonymous with death: *“Before [PEWS], children with cancer would go to the ICU and it was considered a child with no opportunity, that child should die. Once [PEWS] came, our children began to get out and we started saying a child with cancer doesn’t die, we just transfer him too late”* (Nurse, Lima).

### Clinician

In addition to improving patient care, PEWS use led to multiple benefits for *clinicians*, including reduced nursing workload, improved job satisfaction, increased knowledge, and empowerment (**Table 3**).

**Table 3:** Clinician outcomes.

Reduced Nursing Workload	They realized it wasn’t more work, on the contrary, at some point, it would decrease their amount of work (Administrator, Xalapa)
	Thanks to the result of this project, nurses can treat fewer patients (Nurse, Lima)
Job Satisfaction	It’s personally very satisfying to be able to bring that kind of attention to the patients (Nurse, Xalapa)
	The effort that [PEWS] requires is not that big and the satisfaction that we have to prevent a cardiac arrest or death on a patient is much higher (ICU Physician, Cuenca)
	The satisfaction of contributing to my patient’s health, preventing deterioration because my vital signs were taken on time, because my interventions were correct (Nurse, San Salvador)
Knowledge	[Nurses] would take the vital signs … without knowing if the patient was okay or bad until this program was implemented (Ward Physician, San Luis Potosi)
	It helped my knowledge; it expanded my ideas about attention (Nurse, Xalapa)
Empowerment	This situation has helped for the empowerment of the nursing staff, to say hey my job is valuable (Ward Physician, Lima)
	That empowerment, not just from the nursing staff but from the entire multidisciplinary team that participated in the improvement of the patient, has helped with the success of the project (Nurse, San Salvador)

While nurses initially perceived PEWS use as increasing their workload, with continued use, it became part of their workflow, and ultimately, the reduction in deterioration events and earlier PICU transfers due to PEWS was felt to decrease nursing workload as they were caring for fewer critical patients: *“with the implementation of [PEWS] we have patients that stay only a few days at the hospital, less patients at ICU, etc. So they see results, they see less work for them”* (Administrator, Xalapa). Additionally, some centers leveraged the initial increase in nursing workload to advocate for a reduction in the nurse-to-patient ratio: *“Without [PEWS] we couldn’t justify the need of a nurse to take care of 6 children, they used to take care of 10 or 12 before,”* (Nurse, INEN).

As a result of using PEWS to positively impact the care of their patients, clinicians experienced greater job satisfaction: “*you can intervene your patients early and avoid ICU or even death; that gives you great satisfaction,”* (Nurse, San Luis Potosi). Staff members across all disciplines found this to be motivating: *“they see that their work is represented in a patient who is discharged in very good conditions, that makes their effort worthy,”* (Ward Physician, Lima).

Additionally, many staff members, especially nurses, found that PEWS and the accompanied trainings expanded their knowledge-base: “*we used to take signs without knowing what was normal … now with [PEWS], we know how different it is to have a bradycardia or an asymptomatic bradycardia, it changes a lot*,” (Nurse, Cuenca). As a result, they were better able to monitor their patients and felt more confident speaking up when necessary to raise an alarm.

As staff gained knowledge and confidence, they reported increasing feelings of empowerment: “*little by little the nurses found out they could go beyond with their work in the service, more than just give medicine, prepare chemotherapies, the fact that they could evaluate a patient … makes them feel more educated, with more power for decision*,” (Ward Physician, Xalapa). PEWS empowered nurses to take a more active role in patient assessment and management, and physicians felt empowered to contribute to ongoing improvements in patient care: “*there is motivation from the resident part knowing the supervision is higher in the entire service*” (Ward Physician, San Luis Potosi).

### Team

In addition to benefits for patients and clinicians, PEWS led to benefits for the interprofessional *team* including better communication between healthcare providers and improved interprofessional (e.g., nurses and doctors) and interdisciplinary (e.g., ward and ICU) team dynamics (**Table 4**).

**Table 4:** Team outcomes.

Better Communication	Now we’re talking the same language in relation to the patient (Nurse, San Salvador)
	Communication, at the beginning this was a weakness, but then it became a strength (Ward Physician, Lima)
	A lot of benefits regarding the communication between doctors and nurses … even communication with the department of nutrition … the department of physiotherapy (ICU Physician, Cuenca)
Improved Team Dynamics	We saw teamwork which very often is not seen in other units. The involvement of the medical part with the nursing staff and the service staff, with the administrative staff (Nurse, San Salvador)
	The chance to work as a team both with the nurses … the pediatric oncology staff and also the staff at the ICU (Ward Physician, Lima)
	[The doctors] now let us give them suggestions, and before they never heard the observations we told them. (Nurse, San Salvador)

Participants explained that prior to the introduction of PEWS, nurses and physicians did not have common terminology to discuss patient status, resulting in ineffective communication: *“Before [PEWS] … we would come to the doctor and say I can see the patient is getting worse, the patient is not well, but it was subjective; the doctor would say maybe you’re just seeing him that way and maybe you’re wrong,”* (Nurse, Cuenca). PEWS use provided teams a common language, improving interprofessional and interdisciplinary communication: *“with the [PEWS] score, it was so easy with everyone talking about the same thing to detect a patient when he needed to be transferred to the ICU or when they could treat him in the [ward] … now we all speak the same language”* (Ward Physician, San Luis Potosi).

Improvements in team dynamics not only enhanced communication but also improved interprofessional and interdisciplinary relationships. Prior to implementation of PEWS, collaboration between professions was minimal; after implementation, nurses and doctors interacted more frequently and the *“relationship between them improved a lot”* (Ward Physician, Cuenca), *“not only professionally, but friendly”* (Ward Physician, Lima). PEWS diminished hierarchies between physicians and nurses, and as a result, nurses felt their input was welcome and valued in ways that it was not previously: *“that we would all talk the same language and that the nurse would have voice and vote in the evaluation of the patient, it’s been one of the biggest and most successful projects,”* (Nurse, San Salvador). Similarly, the use of PEWS facilitated better relationships between the ward and PICU teams: *“before it was like we must transfer him to the ICU, I’m scared they won’t accept him, but not anymore … they are more sensitized, more accessible,”* (Ward Physician, Lima). Following PEWS implementation, PICU transfers were less chaotic as PICU clinicians were more willing to evaluate and admit patients earlier in the course of illness.

### Institution

Perceived *institutional* impacts of PEWS included cost reduction, a change in hospital culture emphasizing high-quality patient care, receipt of institutional awards, and opportunities for collaboration with other hospitals (**Table 5**).

**Table 5:** Institutional outcomes.

Cost Reduction	We need less resources … we are spending less (Nurse, Cuenca)
	It has resulted in reduction of spending, in hospitalization, in used treatments, the situation of the hospital has been highly improved in that part (Ward Physician, Xalapa)
Emphasis on High-Quality Patient Care	[PEWS] is a strength … moving forward to quality and safety of the patient as well as the institution (Nurse, San Luis Potosi)
	[PEWS] has given us a change in the culture of attention (Nurse, Xalapa)
	This was the example to have bigger or better projects in quality improvement in order to help us with the rest of the processes at the hospital (Ward Physician, Lima)
Awards & Accolades	They gave an award for continued quality improvement from the Ministry (Nurse, San Salvador)
	We were nominated a center of excellence in [PEWS] for Latin America. I think this is one of the biggest achievements, reference for Latin America. (Ward Physician, Lima)
Opportunities for Collaboration	We go outside to train other institutions both in the country and abroad … so [PEWS] grew beyond the hospital and we are very proud as an institution (Ward Physician, Lima)
	They have to come here and we have to go there so we can exchange knowledge and improve every day (Ward Physician, Cuenca)

PEWS use reduced hospital costs by decreasing inpatient days and resource utilization: *“we are spending less; patients arrive in ICU on time and they don’t need a ventilator, vasopressors, they stay in ICU only one or two days … compared to the times when it was too late for them, they would stay a lot of days in ICU, they needed ventilator, expensive medicine … the before and after is remarkable,”* (Nurse, Cuenca).

Additionally, participants at all sites noted that PEWS implementation altered hospital culture to increase emphasis on patient-centered care: *“the culture changed, the culture for the whole medical staff to see the patient in a comprehensive way,”* (ICU Physician, San Salvador). Staff experience using PEWS demonstrated that clinical deterioration was largely preventable, leading to a hospital-wide focus on patient safety: *“[Clinicians] see that his life is in danger or that he could get critical … they visualize that it is very important to be on alert with that patient so he won’t have risks, and applying [PEWS] on all our patients has influenced a lot as part of their safety”* (Ward Physician, Xalapa). Quality improvement projects became more common as staff were inspired to explore other strategies to improve patient care: *“A lot of us have started to get involved in other quality improvement projects that maybe didn’t exist before [PEWS], but it has helped us and pushed us to work … to motivate ourselves as professionals to keep looking for alternatives for our patients,”* (Ward Physician, Lima).

Centers were further motivated by receiving awards honoring their PEWS program from entities such as the Ministry of Health: *“all we wanted was to implement [PEWS] and try to give quality to our patients, but it has been recognized by the Ministry, so that’s an achievement bigger than we expected,”* (Nurse, San Salvador). Additionally, participation in Proyecto EVAT provided new opportunities to collaborate with other hospitals: *“We’re able to visit other countries, know the realities of other people, share experiences, share situations,”* (Nurse, Lima).

### Cycle of reinforcement

PEWS implementation led to benefits for patients, clinicians, teams, and institutions initiating a feedback loop that reinforced ongoing PEWS use (**Figure 1**). Recognition of the patient-level benefits of PEWS led to increased buy-in as clinicians were motivated by opportunities to directly improve patient outcomes: *“It was the motivation of seeing the children who could have had a fatal ending return to the [ward] in a better condition”* (Nurse, Cuenca). Greater job satisfaction and empowerment among staff led to improved interdisciplinary and interprofessional relationships and communication. Hierarchical barriers were reduced and the interprofessional team functioned more cohesively: *“we gained friendship and fellowship, which reinforced our work”* (Ward Physician, Cuenca). As relations improved, so did the work environment, facilitating a change in hospital culture with implications for staff’s wellbeing and patient safety. Additionally, observed reduction in resource utilization and mortality galvanized support for PEWS among hospital leadership: “*Even in the administration field, we can see that if there’s a better response to the patient’s need before he gets critical, this reduces spending and reduces the probability to go to the ICU or get critical or even die”* (Ward Physician, San Salvador). This encouraged leadership support for ongoing staff training and expansion of PEWS within the hospital. Over time, PEWS became embedded in the hospital’s culture and workflow, further reinforcing its continued use: *“The hospital has accepted [PEWS] as part of the staff’s work, so they give us the sheets, they open the doors for the training”* (Nurse, Lima).

## Discussion

Our study demonstrates multiple benefits of PEWS implementation for patients, clinicians, healthcare teams, and institutions and the ways in which these benefits modulate and reinforce one another. Similar to prior studies, we found that staff perceived PEWS to reduce adverse events (8), improve quality-of-care (9), increase staff knowledge, confidence, and empowerment (10), improve interprofessional and interdisciplinary communication (11), and reduce hospital costs (13). Our study, however, additionally demonstrates that improvements in patient outcomes increase staff motivation and job satisfaction, and better interpersonal relationships foster an improved work environment leading to changes in hospital culture including increased emphasis on patient-centered care, patient safety, and quality improvement. The use of qualitative methods allowed for this in-depth exploration of the interplay between the multi-level impacts of PEWS and development of an explanatory model for these impacts as understood by staff directly engaged in PEWS use. Our findings can be used to advocate for PEWS implementation to stakeholders at various levels within an institution by focusing on the outcomes most relevant to them.

Implementation and improvement research is important in resource-limited settings where contextual and infrastructural challenges make implementing evidence-based practices more difficult (14). Correct use of any evidence-based practice is integral to assuring impact, and quality of use must be measured and iteratively improved over time. Process evaluation is a strategy to identify and address gaps at each level of an intervention to maximize implementation success and address barriers to successful use (19, 20). Understanding how PEWS impacts are interrelated helps explain how they are achieved. Our study revealed a cycle of reinforcement which outlines the mechanism by which multilevel outcomes contribute to PEWS success. This process evaluation helps identify critical components of effective quality improvement interventions in settings of all resource-levels, creating a framework for implementation and continuous monitoring (19). Furthermore, understanding impacts relevant to different stakeholders can help address specific barriers and inform targeted and contextually-appropriate strategies to improve intervention adoption and use. This approach can help inform the assessment of PEWS and other clinical interventions.

The cycle of reinforcement identified in our study provides a model to promote the sustainability and expansion (scale) of effective quality improvement interventions. Prior work suggests that an institution’s capacity to sustain an evidence-based practice such as PEWS increases with time (21), and the cycle of reinforcement described in this work identifies a potential mechanism to explain this finding. Furthermore, participants identified components of the clinical capacity for sustainability framework (22), which describes an organization’s capacity to sustain evidence-based interventions across seven domains, including engaged stakeholders, outcomes and effectiveness, implementation and training, and workflow integration (23), as important outcomes of PEWS implementation. More work is needed to prospectively evaluate whether these factors contribute to the maintenance of high-quality PEWS use over time and explore possible strategies to promote sustainability of the multi-level benefits of PEWS use.

Our study has several limitations. Key stakeholder interviews have a risk of social desirability bias (24); however, we attempted to mitigate this by using interviewers previously unknown to participants and not involved in PEWS implementation and by explaining the process of interview de-identification to participants. In addition, interview questions were designed to explore barriers and enablers to PEWS implementation rather than its impacts; identified themes regarding PEWS outcomes were largely spontaneously reported by participants, minimizing bias. All data were collected in Spanish with analysis conducted in English, potentially influencing the interpretation of original statements. To minimize inaccuracies, a professional service was used for translation and 20% of transcripts were audited by a bilingual team member (SG) to confirm accuracy. Finally, this study was conducted in one region (Latin America) among pediatric oncology centers, potentially limiting generalizability of study findings to other regions and patient populations. However, diversity of participating hospitals and similarities between our findings and prior literature on PEWS supports the applicability of these findings to other settings.

This study uniquely describes the interplay between the multilevel impacts of PEWS implementation in resource-limited settings. Benefits at the level of the patient, clinician, team, and institution create a cycle of reinforcement that amplifies impact and supports ongoing PEWS use. These findings can guide advocacy for PEWS to different stakeholders, improve PEWS implementation and efficacy, and inform the implementation and evaluation of other quality improvement initiatives to reduce disparities in childhood cancer outcomes globally.

## Data availability statement

The raw data supporting the conclusions of this article will be made available by the authors, without undue reservation.

## Ethics statement

The studies involving human participants were reviewed and approved by St. Jude Children’s Research Hospital Institutional Review Board. Written informed consent for participation was not required for this study in accordance with the national legislation and the institutional requirements.

## Author contributions

AA and DG developed the idea. MP-T, SG, PE, HM-T, AGR, MA, CB, RDC, CH, SJ, JJL, AM, EMo, EPe, and EPi collected the data. AA and DG provided supervision. EMi, GF, and AA conducted the data analyses. EMi, AA, and DG drafted manuscript and prepared the tables and figures. EMi, GF, and AA had full access to all the data in the study and take responsibility for the integrity of the data and the accuracy of the analysis. All authors contributed to the interpretation of the findings, the editing of the article, and the approval of the final submitted version.

## Funding

This study was funded by the American Lebanese-Syrian Associated Charities (ALSAC). EMi was supported by grant R25CA23944 from the National Cancer Institute. These funders were not involved in the design or conduct of the study; collection, management, analysis, or interpretation of the data; preparation, review, or approval of the manuscript; or decision to submit the manuscript for publication.

## Acknowledgments

We thank the PEWS implementation teams at all Proyecto EVAT centers, including those who participated in this study, as well as the Proyecto EVAT Steering Committee for oversight of this work.

## Conflict of interest

The authors declare that the research was conducted in the absence of any commercial or financial relationships that could be construed as a potential conflict of interest.

## Publisher’s note

All claims expressed in this article are solely those of the authors and do not necessarily represent those of their affiliated organizations, or those of the publisher, the editors and the reviewers. Any product that may be evaluated in this article, or claim that may be made by its manufacturer, is not guaranteed or endorsed by the publisher.
